# Combining statistical and dynamical modelling to guide the design of cluster randomised trials for malaria

**DOI:** 10.1136/bmjgh-2026-024344

**Published:** 2026-08-28

**Authors:** Joseph D Challenger, Joseph Biggs, Janetta Skarp, Jackie Cook, Thomas S Churcher

**Affiliations:** 1 Medical Research Council Centre for Global Infections Disease Analysis, Department of Infectious Disease Epidemiology, School of Public Health, Imperial College London, London, UK; 2 International Statistics and Epidemiology Group, Department of Infectious Disease Epidemiology and International Health, London School of Hygiene & Tropical Medicine (LSHTM), London, UK

**Keywords:** Global Health, Epidemiology, Public Health, Cluster randomized trial

## Abstract

Cluster randomised trials (CRTs) remain key for evaluating the community-wide impact of interventions against infectious diseases such as malaria. Randomising by cluster prevents contamination and enables both the direct and indirect effects of the intervention to be estimated. Although these trials are extremely informative, they can be logistically demanding and costly to carry out, which means it is important that these trials are well powered. Here, we present a framework for planning CRTs that measure malaria prevalence as the outcome using an established mathematical model of malaria transmission. In this way, we explicitly consider the epidemiology of the individual trial clusters. The framework can be used alongside a baseline prevalence survey to help inform the sample size calculations for the trial. We use a case study to illustrate the framework, where we simulate a CRT in which a next-generation pyrethroid-pyrrole insecticide-treated net (ITN) is compared against a standard pyrethroid-only ITN. We show how the malaria endemicity of the trial location and timing of the follow-up surveys can affect the results obtained. We also highlight how other active interventions against malaria can reduce study power by increasing the amount of between-cluster heterogeneity in malaria prevalence.

**WHAT IS ALREADY KNOWN ON THIS TOPIC**

- Cluster randomised trials (CRTs) are the gold standard for evaluating the community-level impact of novel control tools against infectious diseases such as malaria.
- However, these trials are logistically demanding and expensive to carry out, which means that it is important that these studies are well powered to detect a difference in measured outcomes between arms, if a true difference exists.

**WHAT THIS STUDY ADDS**

- We have developed a modelling framework that combines statistical and dynamical modelling to enable modelling predictions to be made for CRTs that measure malaria prevalence as an endpoint.
- The framework highlights how the effect size of an intervention and the between-cluster heterogeneity in malaria prevalence can change over the course of a trial, affecting statistical power.

**HOW THIS STUDY MIGHT AFFECT RESEARCH, PRACTICE OR POLICY**

- This framework can be used once the trial organisers have carried out their baseline prevalence survey, and could inform the timings of the follow-up prevalence surveys and the sample sizes to use.

## Introduction

 Cluster randomised trials (CRTs) remain the gold standard for evaluating the community-level impact of novel control tools against infectious diseases.^1^ For malaria, this methodology has been used to assess the impact of interventions such as insecticide-treated nets (ITNs), indoor residual spraying (IRS), and mass drug administration.^2^ Although these trials form an important part of the evidence base for the efficacy of an intervention, they are time-consuming, logistically demanding and costly to run. Randomising by cluster, rather than by individual, has the effect of inflating the sample size required to adequately power a trial.^1^ This inflation factor, known as the ‘design effect’, is driven by the between-cluster heterogeneity in the measured outcome. This heterogeneity should be measured or estimated prior to the intervention rollout in order to estimate the required sample size (the number of clusters per arm and the number of people to be sampled in each cluster). A recent systematic review of malaria CRTs showed that only a minority of trials used data to guide their prediction of the between-cluster heterogeneity.^2^ Furthermore, empirical estimates of this heterogeneity measured from trial data often exceed the values used in the sample size calculations meaning that many of the studies were underpowered.^3^

Another key parameter in the sample size calculation is the effect size that the novel intervention is expected to achieve. In many trials, the selected effect size is a compromise between being small enough to be realistic, but big enough to make the logistics of the trial feasible. Clearly, the effect size will depend on the characteristics of the intervention in question, but it will also depend on the coverage level of the intervention achieved during the trial and the intensity of transmission in the trial sites. It could also vary with the outcome metric used and the characteristics of the population studied (eg, age of the study population). Estimating the epidemiological effect size of a new intervention is often non-trivial. The malaria transmission cycle is complicated^4^ and a typical intervention will only target certain elements. Potential targets include: reducing mosquito densities by killing adult mosquitoes (*via* vector control tools such as insecticide-treated nets or indoor residual spraying), reducing the infectiousness of the human population to a feeding mosquito (*via* mass drug administration or transmission-blocking vaccines), or reducing the ability of mosquitoes to enter homes *via* housing modification, thereby reducing the biting rate. Mechanistic transmission models can be helpful as they are able to estimate the relationship between an intervention’s impact on the relevant component (or components) of the transmission cycle, the intervention coverage across a population and the overall epidemiological impact.

During the course of a trial, secular changes in transmission could occur (eg, through environmental change) or the standard of care used in both arms may change, which could modulate the apparent impact of the novel intervention. In many locations, disease transmission is highly seasonal,^5^ which can mean that the measured impact of a novel intervention changes over time, even if the intervention is still performing well. A recent meta-analysis of malaria CRTs^3^ reported higher between-cluster heterogeneity at lower malaria endemicity. This suggests that an effective intervention could increase the coefficient of variation in the corresponding trial arm, potentially diminishing study power. There is a need for more considered estimates of the effect size and the coefficient of variation to ensure that future trials are adequately powered to ensure limited resources are not wasted.

An additional challenge is that, in many areas, a novel intervention is usually layered on top of existing interventions against the targeted disease. This can reduce the effect size that the new intervention can be expected to have.^2^ The use of other interventions may also have implications for the age group selected for follow-up in the trial. For example, if young children are receiving seasonal malaria chemoprevention (SMC),^6^ it may be harder to measure the impact of a new intervention by following this age group.

In this work, we propose the use of a widely used and validated mechanistic transmission dynamics mathematical model of malaria to aid the CRT planning process. We illustrate our methodology for CRTs that measure malaria prevalence as the primary endpoint. Using the example of an unmatched, two-arm CRT with a parallel design, we will examine how factors such as malaria endemicity, the age group selected and the presence of other active interventions can affect both the expected effect size for the trial and the between-cluster heterogeneity. In this way, we will show the ensuing consequences for study power. We will then show how the modelling pipeline could be used to help trial planning, using cluster-level information collected at baseline. We show modelling results for several trial scenarios and discuss the consequences for using modelling for informing CRTs.

## Results

### Modelling framework

**Figure 1:**
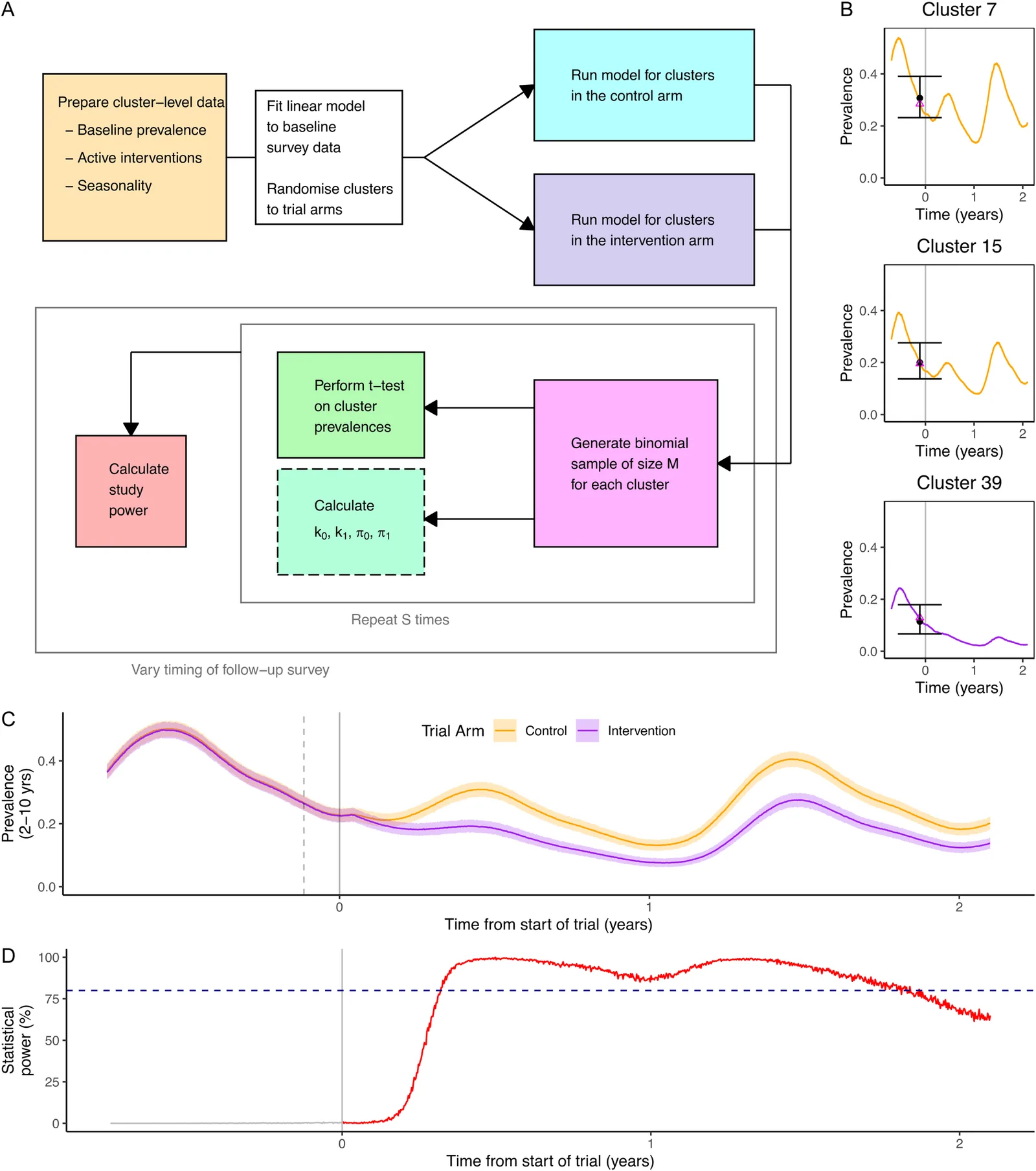
The framework for modelling cluster randomised trials. Panel A: To contextualise malaria transmission in a given cluster, we use information about seasonality of transmission and active interventions against malaria. The prevalence data at baseline (simulated or empirical) is used to calibrate the transmission model for each cluster, after a linear mixed-effects model is used to account for the sampling variation (see the Methods section). In the calibration process, we vary the transmission intensity to match the desired prevalence, given the interventions in place. Clusters are then randomised to the trial arms. For each cluster, the transmission model is run for the trial period. To generate a follow-up prevalence survey at a chosen timepoint, a binomial sample of size m is generated using the model-predicted prevalence in each cluster. An unpaired *t* test is performed to assess whether a significant difference is detected between trial arms. Statistical power is estimated by repeating the sampling process S times, each time also estimating the coefficient of variation (*k_0_* in control clusters, *k_1_* in the intervention clusters) and mean cluster prevalence (*π_0_* in control clusters, *π _1_* in the intervention clusters). Panel B shows the cluster-level model runs for three illustrative clusters (black circles are the raw prevalence values, magenta diamonds are the prevalences predicted by the linear model). Panel C shows how the arm-level prevalences evolve over the course of the trial. Panel D shows an estimate of the study power (*S*=1000) for each day of the trial period.

The modelling framework used throughout this work is depicted in figure 1 and is described in detail in the Methods section. The framework can be used alongside an empirical baseline prevalence survey to help inform adaptations to sample size estimations for the follow-up surveys in the CRT. Equally, the framework could be used earlier in the trial planning process to help determine the type of setting in which to carry out the CRT. In this work, we will use synthetic (simulated) baseline surveys to demonstrate the methodology, with c clusters in each arm. These surveys incorporate between-cluster heterogeneity, which we quantify in terms of the coefficient of variation (k) in the cluster-level prevalences (see the Methods section). Clusters are assigned to the treatment arms using restricted randomisation. Once the transmission modelling has been carried out for the trial period in each cluster, simulation-based methods are used to estimate trial power (see the Methods section). In this work, we generate estimates for statistical power for each day of the trial, that is, as though prevalence surveys were conducted every day in each cluster. Clearly, this is not feasible in practice, but it allows us to explore how statistical power varies over the course of a trial and when prevalence surveys might be conducted in a real study.

We now illustrate the framework with a hypothetical example. We consider a cluster-randomised trial to evaluate pyrethroid-pyrrole ITNs compared with pyrethroid-only ITNs over a 2-year period, in a setting with seasonal malaria transmission and moderate levels of pyrethroid resistance. In the study region, 40 clusters have been identified, which will be randomised 1:1 to each study arm (c=20). The cluster-level prevalences at baseline, in children aged 2–10 years, are sampled from a distribution with an average prevalence of 0.3, with k=0.3. Historically, ITN coverage has been about 60% in the region: during the trial, it is hoped that 80% coverage is achievable immediately following the mass ITN campaign. In the trial, it is expected that at least a 30% relative reduction in prevalence can be achieved using the next-generation ITNs, compared with the pyrethroid only arm (ie, an absolute reduction from 0.3 to 0.21). Given the number of clusters to be used, the baseline prevalence, between-cluster heterogeneity and expected effect size, we calculate that a sample size of 50 people per cluster per survey will be sufficient for 80% power (at the 5% significance level).

**Figure 2:**
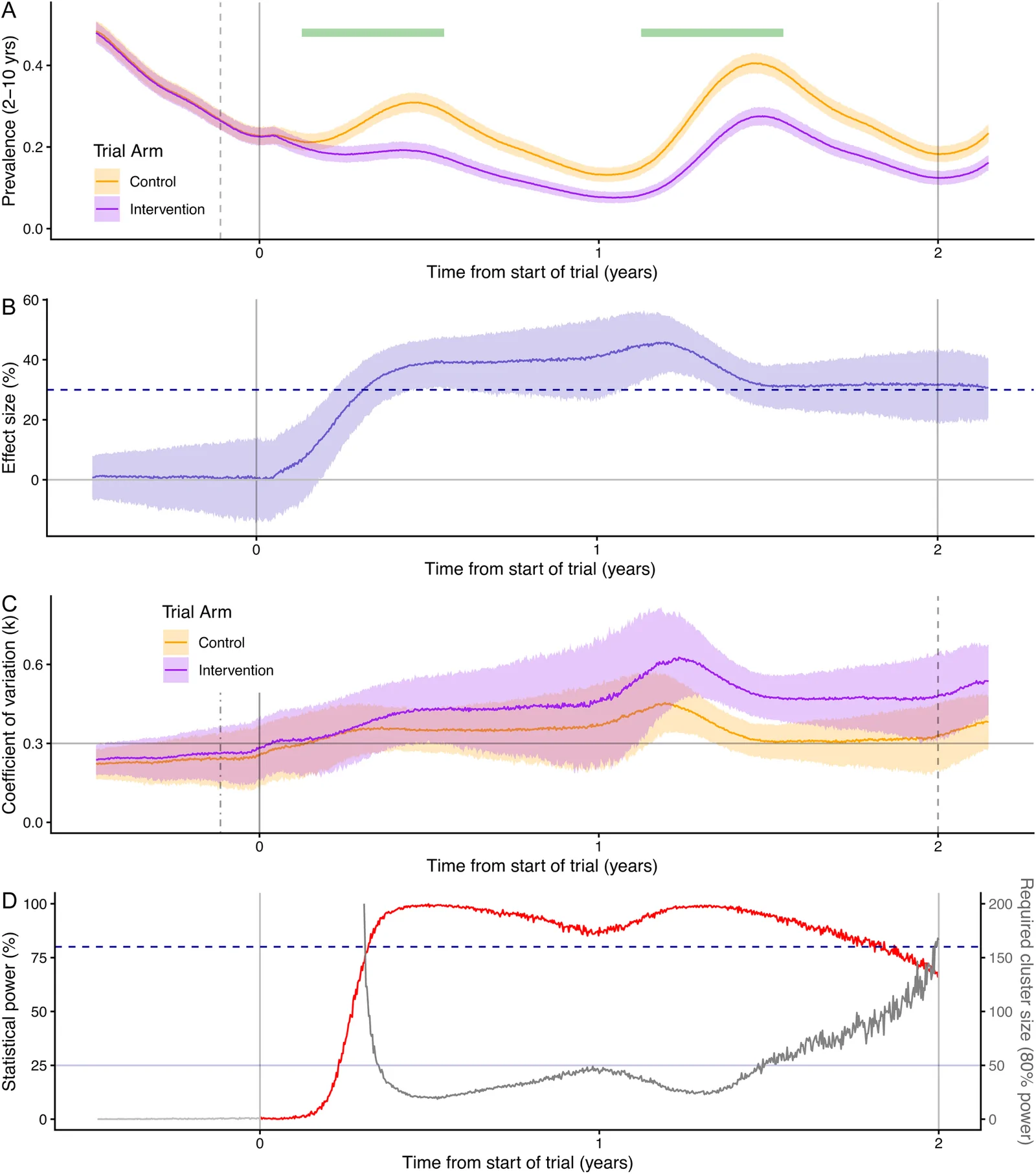
Modelling output generated for a cluster-randomised control trial (CRT). Here, we show simulated output for a malaria CRT evaluating a next-generation pyrethroid-pyrrole insecticide-treated net (ITN) against a standard pyrethroid-only ITN. A baseline prevalence survey was simulated for a trial with a between-cluster coefficient of variation of 0.3 and an average malaria prevalence of 0.3. Clusters were randomly assigned to the two trial arms, with 20 clusters per arm. A seasonal setting was selected for the trial, with the horizontal green bars in panel A indicating the malaria season in each year of the trial period. After the malaria transmission model was simulated for each cluster, follow-up surveys for a given time point were generated using a sample size of 50 in each cluster. Estimates for the arm-level prevalences (panel A), the effect size (panel B, horizontal dashed line indicating a 30% effect size), the between-cluster coefficient of variation in each arm (panel C, grey vertical dot-dashed line indicating the timing of the baseline survey, relative to the start of the trial) and statistical power (panel D, horizontal dashed line indicating 80% power) were generated from the cluster-level prevalence surveys. In each panel the shaded areas represent the 95% range for the variation generated from simulating 1000 follow-up surveys on each day of the follow-up period. We note that power remains high for much of the 2-year trial, between months 4 and 22 of the trial, before decreasing towards the end of the trial. Panel D also shows (grey line, secondary y-axis) the sample size per cluster required to obtain 80% power, using the central estimates for the quantities shown in panels A and C and Equation 1 (see the Methods section). The solid blue horizontal line marks a sample size per cluster of 50, used for the initial sample size calculations.

In figure 2, we show model-predicted outputs for this scenario. We display the time evolution of the arm-level prevalences, the effect size due to the trial intervention and the between-cluster heterogeneity, as these all influence the statistical power. In this example, high statistical power is achieved for much of the trial period (from month 4 to month 22 of the trial), before decreasing towards the end of the study. We note here that there is quite high variability in the values of k obtained, indicating that k is quite sensitive to the sampling variation generated by the prevalence surveys. We also see higher values of k in the intervention arm, as prevalence is reduced. In figure 2D, we show (secondary y-axis) the sample size per cluster required for 80% power over the course of the study, based on the central estimates of k and prevalence in each arm, as predicted by the modelling (see the Methods section). Early in the trial, when the effect size is small, we see that 80% power cannot be reached in this study, with the current number of clusters.

### Disease endemicity and seasonality

When planning a CRT, a decision must be made as to where it should be carried out. Part of this decision can involve choosing whether to evaluate the novel intervention in a high-transmission, moderate-transmission or low-transmission setting. The modelling framework can help to explore how varying the average endemicity of the study region can affect the predicted trial results. We consider a similar trial to the one presented in the previous subsection, now varying the average baseline malaria prevalence from 10% to 45%, again in children aged 2–10 years. In practice, all-age prevalence is more likely to be used in low-transmission sites, but we keep the age group fixed here, for comparability across endemicity levels. We show results for both a seasonal and non-seasonal setting, presenting outputs at eight timepoints during the 2-year trial, as illustrated in online supplemental figure 3.

It is important to note here that varying the endemicity in this way has several implications for a CRT. First, it will affect the sample size required for a desired value of statistical power for a fixed relative reduction (see Equation 1 in the Methods section). In addition, recent work^3^ has shown that higher values of k have been observed in malaria CRTs carried out at lower endemicity. Finally, in low-prevalence settings, it is reasonable to expect a larger effect size in relative terms, to consider an intervention impactful (eg, a reduction from 10% to 5% is a large relative reduction, but a fairly small one in absolute terms). Therefore, in the main text of the article we present scenarios in which k changes with endemicity and in each scenario the number of clusters per arm, c, is chosen to keep statistical power at 80% (see online supplemental table 1). At low endemicity (prevalence <20%), we also show results obtained when larger effect sizes are expected (with c recalculated to ensure 80% power, see online supplemental table 1).

**Figure 3:**
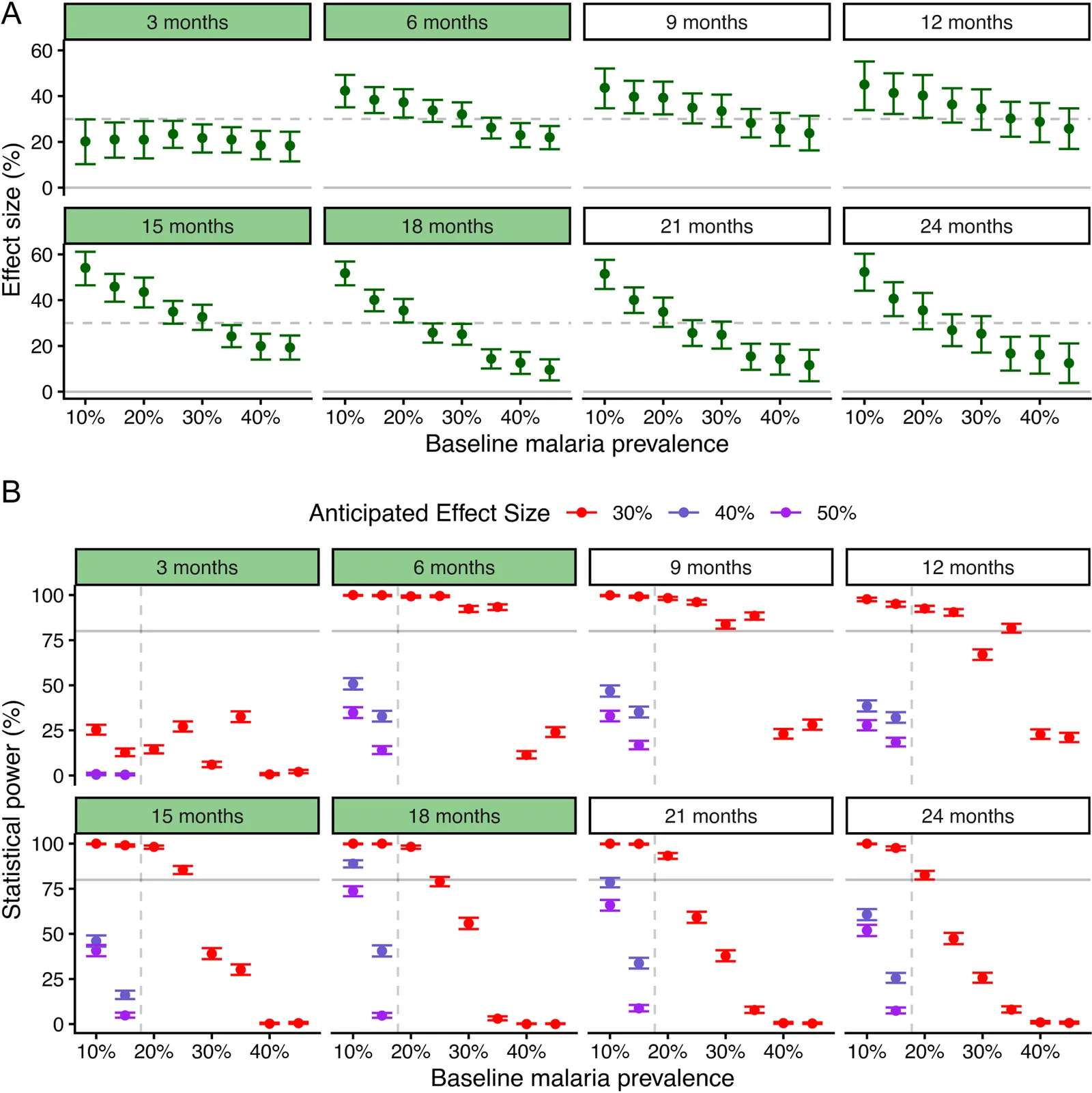
Estimating trial outcomes at different levels of malaria endemicity in a seasonal setting. Here, simulated trial data is assessed at eight different follow-up times, in a trial lasting 2 years (scenario is illustrated in online supplemental figure 3B). The mean prevalence in the baseline survey (measured in 2–10 year olds) is varied from 10–45%. Panel A: Time evolution of the achieved effect size at different malaria endemicities (x-axis). In low-transmission settings, we see that a large effect size is established by the end of the first malaria season (see panel labelled ‘6 months’), and that this persists for the duration of the 2-year trial period. At higher transmission intensity, the effect size is smaller and reduces towards the end of the trial period. This observation is reflected in the power estimates (panel B), indicating that it is difficult to achieve good study power in high transmission settings for the sample sizes chosen here. At intermediate transmission intensity (baseline prevalence of 20%), >80% power is maintained until the end of the 2-year period. At low transmission intensity, we also show results obtained when the expected effect size (relevant for the sample size calculations) is expected to be >30% (see legend of panel B). Study power is low in these scenarios, in part because the between-cluster heterogeneity becomes elevated, relative to the baseline values (see figure 4). The sample size used in each scenario is shown in online supplemental table 1. In both panels A and B, measurements taken during the malaria season are highlighted with the light green shading.

**Figure 4:**
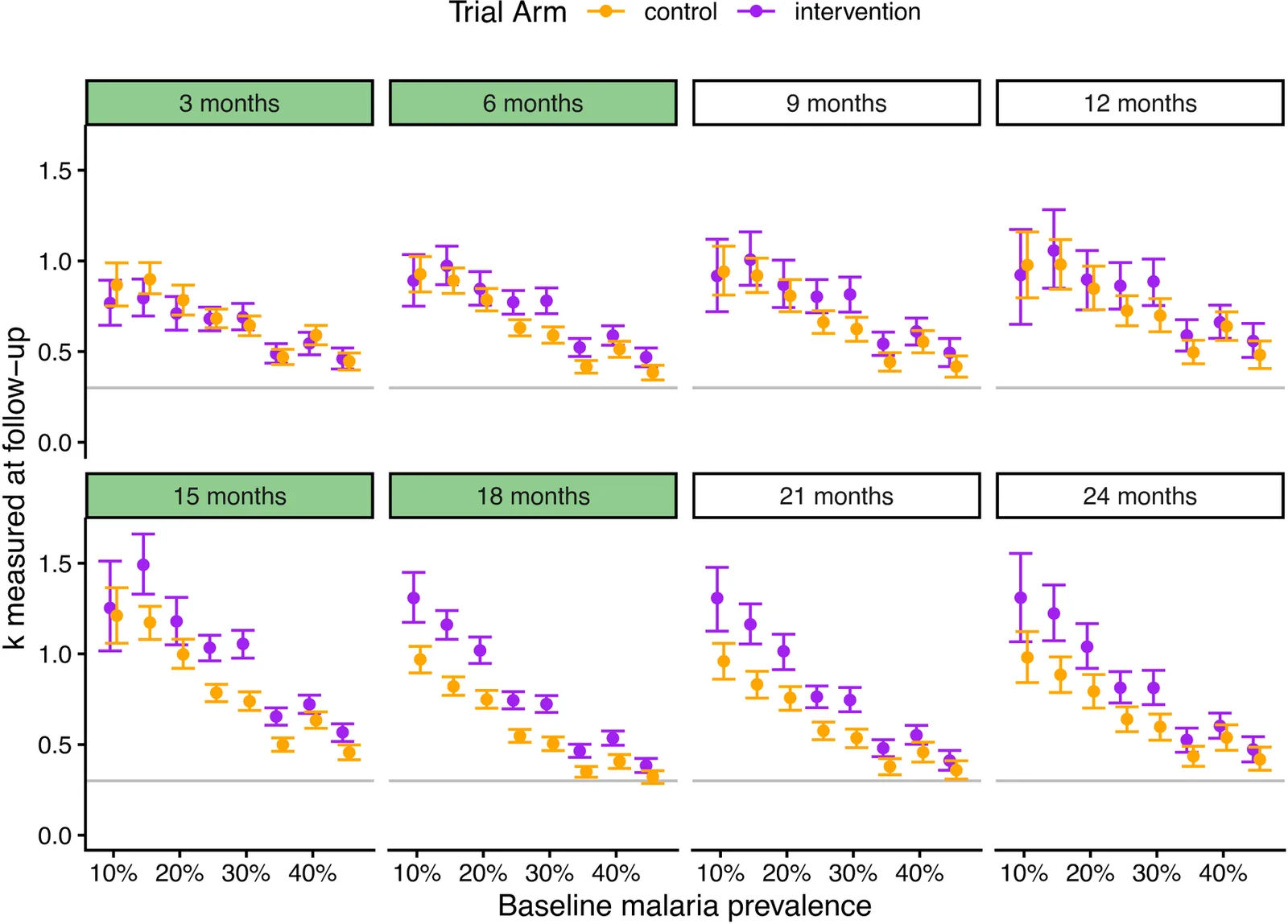
Estimating the between-cluster heterogeneity (*k)* at different levels of malaria endemicity in a seasonal setting. Here simulated trial data is assessed at eight different follow-up times, in a trial lasting 2 years (scenario is illustrated in online supplemental figure 3B). The average prevalence at baseline is varied from 10–45% (in 2–10 year olds). The value of *k* at baseline varies with prevalence, as shown in online supplemental table 1, with higher values used at lower prevalence. Three months into the trial, the average values of *k* (points) are similar in each arm. Later in the trial, however, *k* increases as prevalence decreases, especially in the intervention arm. The very high values of *k* observed in low transmission settings towards the end of the trial period make it harder to power these studies, especially when a large effect size was expected (figure 3B). The error bars indicate the central 95% range of estimated *k* values at each time point, generated by simulating 1000 follow-up surveys. The horizontal grey line (*k*=0.3) is shown as a reference. Measurements taken during the malaria season are highlighted with the light green shading.

Figure 3 shows results for the predicted effect size and study power in the setting with seasonal transmission. In online supplemental figure 4, similar results are shown for the setting with perennial malaria transmission. The statistical power is influenced not only by the achieved effect size, but also by the between-cluster heterogeneity. We show the time evolution of k in figure 4 (seasonal setting) and online supplemental figure 5 (non-seasonal setting). In the seasonal setting, the trial starts just before the malaria season, with around 90% of the year’s malaria transmission occurring in months 2–6 of the trial. Due to this intense period of transmission, we find that statistical power rises more quickly here (figure 3) compared with the non-seasonal setting, in which transmission is spread evenly over the whole year (online supplemental figure 4). In both the seasonal and non-seasonal cases, in high-transmission settings we see that the effect size diminishes towards the end of the trial period, leading to a reduction in statistical power. We expect this pattern to be characteristic of ITNs, as insecticide concentrations wane over time and individuals may cease using their net if it becomes damaged. In low-transmission settings, the effect size remains high through the second year of the trial period. However, due to the higher between-cluster heterogeneity at baseline (online supplemental table 1), these scenarios require a much larger sample size to power. Furthermore, the transmission modelling suggests that the between-cluster heterogeneity increases over the course of the trial (online supplemental figures 4 and 5) as prevalence is reduced. This is consistent with the finding in previous work,^3^ where higher values of k were found in surveys where the average prevalence was lower. This means that even though the measured effect size remains high in low-transmission settings, the elevated between-cluster heterogeneity (ie, above the value measured at baseline) can still lead to the study being underpowered. These modelling results predict that the CRT considered here is easier to power at intermediate values of malaria prevalence (around 20%), particularly in the seasonal setting.

In the analysis presented above, multiple factors (endemicity, sample size, between-cluster heterogeneity) are changing at the same time. For comparison, we also show what happens when endemicity is varied while the sample size and baseline k values are held fixed (online supplemental figure 6A for the seasonal setting and online supplemental figure 7A for the non-seasonal setting). As changing the endemicity affects the sample size required for the desired value of statistical power, we also look at the intermediate case, where k is held fixed, c is selected to maintain 80% power (see online supplemental table 2), and higher effect sizes are anticipated in low-transmission settings (online supplemental figure 6B for the seasonal setting and online supplemental figure 7B for the non-seasonal setting). The most noticeable difference between these results and the scenarios presented in the main text is that lowering prevalence but fixing k raises power above 80% for an expected effect size of 40%, although power is still below 80% when the expected effect size is 50%. In online supplemental table 2, we also see that the required sample sizes are lower here, compared with the case where k varies with endemicity (online supplemental table 1). However, given the findings presented in previous work,^3^ we stress that we believe that the results presented in the main text, in which k varies with endemicity, are the most realistic.

### Background interventions

CRTs for malaria will be conducted in settings with other ongoing public health interventions, designed either to reduce malaria transmission, protect vulnerable individuals or both. It would be useful to consider how these ‘background’ interventions could affect a CRT, particularly if they are not conducted in all study clusters. The background intervention we will use as an example here is SMC, which is typically given to young children during the malaria season.^6^ In a country or subnational region, the decision on whether to administer SMC is influenced by multiple factors, but an important consideration is the seasonality of transmission e.g. the percentage of annual malaria incidence that occurs within four consecutive months of the year^5^ In some countries (Ghana, for instance), some regions would qualify for SMC under this guideline, while others would not. As before, we design a trial to evaluate a pyrethroid-pyrrole ITN against a standard pyrethroid-only ITN. In half of the study clusters, children aged between 3–59 months will receive four doses of sulfadoxine-pyrimethamine plus amodiaquine (SP-AQ) during the malaria season. To mitigate against this, it is decided in the trial planning stage to balance the number of SMC and non-SMC clusters during the restricted randomisation procedure, as well as balancing the clusters on their average prevalence.

**Figure 5:**
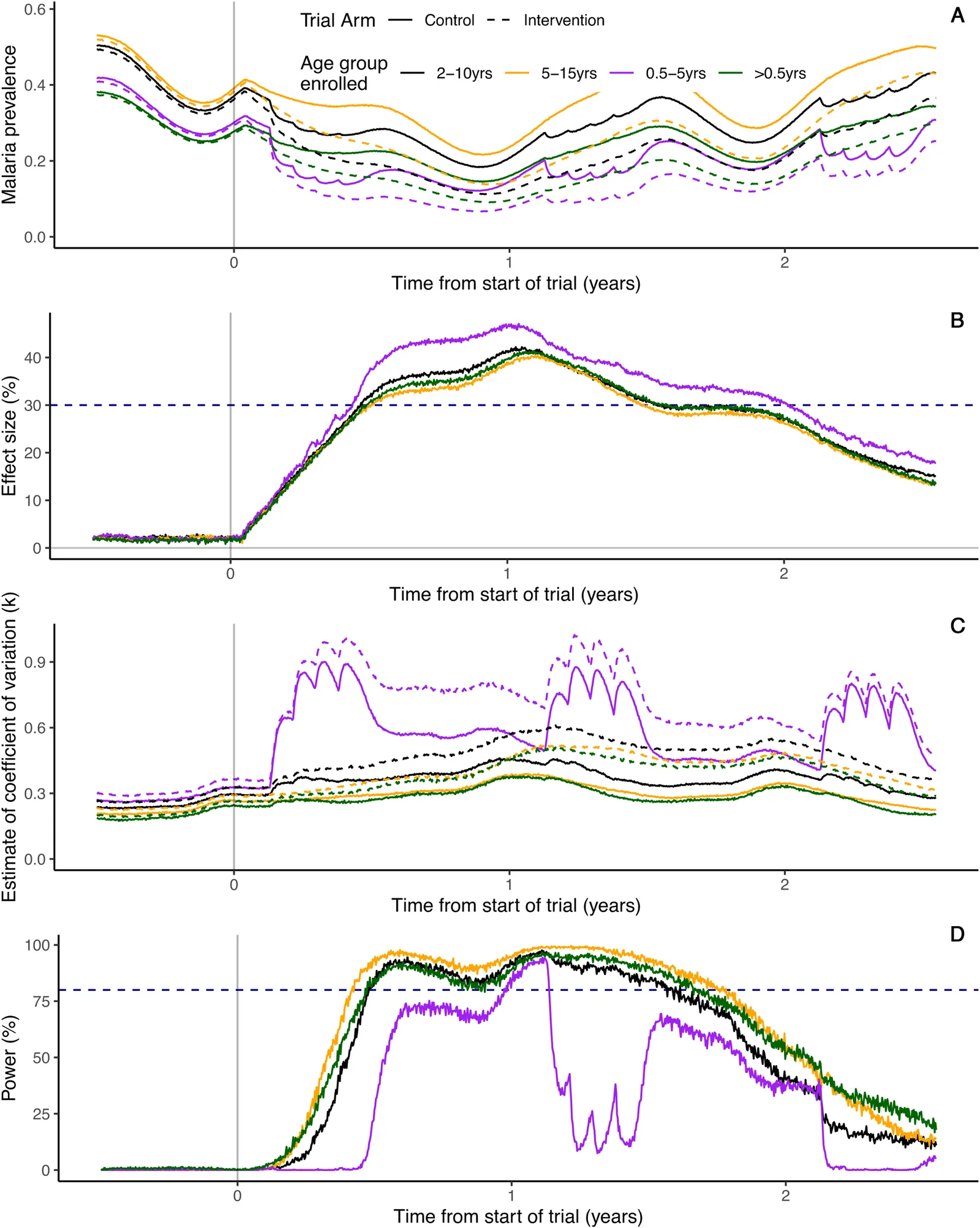
The impact of background interventions on cluster randomised trials (CRTs). In this simulated CRT, we evaluate a pyrethroid-pyrrole insecticide-treated net (ITN) against a standard pyrethroid ITN. In half of the clusters, children aged 3–59 months receive seasonal malaria chemoprevention (four rounds of sulfadoxine-pyrimethamine plus amodiaquine (SP-AQ)) during the transmission season. Although the number of seasonal malaria chemoprevention (SMC) clusters is balanced between the arms, we find that between-cluster heterogeneity is greatly increased if follow-up surveys are conducted in the age group that receive SMC. Panel A: Arm-level malaria prevalence in four different age groups: 2–10 years (black), 5–15 years (yellow), 0.5–5 years (purple), all ages above 0.5 years (dark green), with the solid line showing the control arm and the dashed line the intervention arm. Panel B: Effect size of the trial intervention over time in the different age groups. The effect size is largest in the youngest arm group. Panel C: The between cluster variation over time, in the four age groups. Due to the use of SMC in some of the study clusters, we see that between-cluster heterogeneity greatly increases in the 0.5–5 years group. Panel D: Statistical power over the course of the trial. Here, we see that the elevated between-cluster heterogeneity reduces power when children aged 0.5–5 years are followed-up, despite the fact that the relative effect size is highest in this age group. Results were generated using 1000 simulations, with mean values of each measurement presented.

An advantage of our framework is that we can also examine study power for trials following different age groups. This is relevant here as the SMC intervention, present in only some of the clusters, will have a significant impact on prevalence in the target age group (provided that SMC coverage is high), and only a small, indirect impact on the rest of the population (due to a reduction in the infectious reservoir). Figure 5 shows simulated trial results for four different age groups. In the youngest of the four age groups (0.5–5 years), we see a dramatic increase in the between-cluster heterogeneity introduced by the SMC which causes the statistical power to fall dramatically. However, this effect is not visible when follow-up includes older age groups: this finding is intuitive, as the youngest age group overlaps almost completely with the target age group for SMC. It is interesting that this effect is not visible for the 2–10 years old group, where a minority of children (~40%) receive SMC. Therefore, for a trial of this type, it might be advisable to conduct the follow-up surveys in older children, rather than the SMC-eligible population. When children aged 5–15 years are used for follow-up, >80% power is maintained from month 4 to month 21 of the trial period (figure 5). However, if it were decided that it was important to quantify the impact of next-generation ITNs in the youngest age groups (eg, because this age group is particularly vulnerable to clinical malaria), the modelling results presented here could suggest that the trial should be conducted only in regions receiving (or not receiving) SMC to reduce between-cluster heterogeneity. As a sensitivity analysis, we also looked at the case where the 0.5-to-5-year-old age group were used for follow-up and the analysis was adjusted for whether SMC was used in each cluster.^1^ This did improve study power, but power was still <80% for most of the trial period (online supplemental figure 8).

Although this example is a specific one, it demonstrates how our modelling framework can be used to explore various scenarios, and shows how conducting follow-up surveys in different age groups can affect trial outcomes. It is important to note that, in this hypothetical example, the trial organisers knew which clusters were due to receive SMC and could balance the clusters accordingly. Had this not been known, it would have been likely that the number of clusters receiving SMC would not have been balanced across the treatment arms, which would have biased the trial results.

## Discussion

In this work, we have introduced a framework for simulating malaria CRTs that use prevalence as the primary endpoint and provided some simple examples, to illustrate how the modelling framework could be used to guide trial design. We have focused on trials with an unmatched parallel design, as this design was the one found to be used the most in a recent systematic review of malaria CRTs. The framework can be used with either a simulated baseline survey (as done here), or real survey data. Varying both the number of clusters and the sample size per cluster will influence the cost and personnel needed to implement the trial. It is useful, therefore, to vary both and explore the consequences for statistical power.

Although we have focused on next-generation ITNs as the new intervention to be evaluated, in principle any novel intervention could be used. The only requirement is a quantification of how the intervention impacts the malaria transmission cycle. For example, if the novel intervention were a drug (or drug combination) for mass-drug administration, one would need to specify the treatment’s efficacy at clearing existing infections and protecting against incident infections, and how the latter quantity changes with time since administration.

There are many factors to consider when deciding on a trial design. The modelling results presented here do not reflect the challenges of conducting a real CRT, for example, delivering new interventions on time across a wide geographical area can be challenging, prevalence surveys will not be conducted synchronously across clusters, and it may be difficult to ensure high intervention coverage in all clusters. In that sense, the results presented here can be seen as an idealised prediction of a CRT. Despite these simplifications, we feel there is utility to see where study power could be low, even under idealised conditions.

This study highlights several factors that researchers should consider when selecting a location for a future trial. For example, it demonstrates that statistical power tends to be greater in areas with intermediate baseline prevalence. It can be hard to power studies in low-transmission settings, if there are few transmission events (ie, new malaria infections) to detect. On the other hand, using prevalence as an endpoint in a high transmission setting may make it more difficult to measure impact, due to the presence of superinfections. However, we point out that successful malaria CRTs have been conducted in high transmission settings (see for example previous works^7 8^). The seasonality of transmission in a given setting and the duration of an intervention’s efficacy may both inform the timing of follow-up surveys and their sample size. Lastly, differences in the use of alternative control measures, illustrated here using SMC, can diminish study power even if the use of these interventions is balanced between study arms. While many of these considerations are intuitive, the framework presented here provides a systematic approach to formalising and communicating these intuitions. Nevertheless, care should be taken interpreting study results. All power estimates rely on model prediction which are no substitute for empirical data from well-designed trials. The malaria model presented here makes many structural and parameter assumptions and could influence power estimates. It is also important to tailor power calculations to the local context and the type of novel control tool under consideration. Bespoke trial simulations could be undertaken to support trial design. Alternatively, a wider selection of settings could be simulated to explore broad patterns that could improve study precision.

The work presented here is not the first attempt to use transmission modelling to plan CRTs. In malaria, modelling-derived estimates for the effect size of a novel intervention have been used to inform CRTs. An example of this is modelling projections for ivermectin,^9^ which have been used to inform multiple CRTs (see for example previous works^10 11^). Regarding other pathogens, modelling work has been used to inform design of CRTs for Ebola vaccines,^12 13^ soil-transmitted helminths^14^ and HIV prevention campaigns.^15^ Silkey *et al* proposed a methodology for planning a CRT with a stepped-wedged design which they illustrated using a generic SIS model.^16^ However, in these papers we do not see the holistic framework outlined here (figure 1), where the transmission model is parameterised and run at the cluster level and the uncertainty from the measurement process (which here represents the follow-up prevalence survey) in each cluster is propagated through to the hypothesis-testing stage. In our work, the between-cluster heterogeneity is not a static parameter: rather it is a dynamic quantity which varies over the course of the trial, thereby influencing the trial outcomes. Therefore, this work could have applicability beyond malaria to other infectious diseases, with our statistical framework being used alongside a transmission model for a different pathogen.

There are a number of avenues for future work, including the validation of the framework against historical CRTs. Our framework allows a great deal of cluster-level information to be incorporated into the modelling. It would be interesting to explore how variation in quantities such as intervention coverage or pyrethroid resistance would affect study power. CRTs that stratify clusters can greatly reduce between-cluster heterogeneity but introduce a trade-off between reduced heterogeneity and reduced statistical df.^1^ Modelling projections could provide insight into this trade-off, for a specific research question. It would be interesting to consider trials that use malaria incidence as an endpoint, and the role that the type of case detection employed has on the sample size required to power the trial.

CRTs play an important role in evaluating new control tools for infectious diseases, such as malaria. They benefit from a rigorous statistical foundation, which we have not sought to improve on here. Dovetailing the statistical foundation with a dynamical modelling framework allows us to examine how the impact of a control tool varies over time and at different transmission intensities. We hope that using the statistical and dynamic modelling together, in the way outlined here, can help guide CRT planning in the future.

## Methods

### Trial outline

We shall begin this section by outlining a hypothetical trial scenario. A two-arm trial has been planned with 2c clusters, that is, c clusters in each arm. A baseline survey has been carried out in each cluster, which will be used to help randomise clusters to the trial arms and guide the sample-size requirements for the follow-up prevalence surveys. Any additional information used for restricted randomisation of clusters should be introduced at this stage.

A key factor in planning a CRT is estimating the amount of between-cluster heterogeneity present in the trial.^1^ This is commonly quantified by the coefficient of variation (k=σ/π), the SD of cluster prevalences (σ) divided by the mean cluster prevalence (π). To underline the impact that k can have, we state the standard formula for the sample size of an unmatched, two-arm CRT^1^:


(1) $$c=1+\frac{{\left(z_{\alpha /2}+z_{\beta }\right)}^{2}\left(\frac{\pi_{0}\left(1-\pi_{0}\right)}{m}+\frac{\pi_{1}\left(1-\pi_{1}\right)}{m}+k_{0}^{2}\pi_{0}^{2}+k_{1}^{2}\pi_{1}^{2}\right)}{{\left(\pi_{0}-\pi_{1}\right)}^{2}}.$$

Here, the formula is written in terms of the number of clusters per arm (c) required for a trial with 100(1-β)% power of obtaining a significant difference between trial arms (p§amp;lt;α, for a two-sided test), with the true average prevalences of $\pi_{0}$ and $\pi_{1}$ in the control and intervention arms respectively. Here, $z_{\alpha /2}$ and $z_{\beta }$ are z scores, corresponding to upper tail probabilities, for the standard normal distribution. Throughout this work, we will consider outcomes with p§amp;lt;0.05 as significant. The formula uses a sample size of m in each cluster for the follow-up survey, with $k_{0}$ and $k_{1}$ being the between-cluster coefficients of variation in the control and intervention arm respectively. The presence of the between-cluster variation inflates the number of clusters (and thereby the overall sample size) required to power the trial. Frequently, it is assumed that $k_{0}$ and $k_{1}$ can be set to the same value, which assumes the intervention has the same relative effect across clusters. However, we will treat them as distinct quantities here.

In the online supplemental methods, we outline the methodology used to estimate the cluster-level prevalences used for the transmission modelling. This is also depicted in online supplemental figure 1. We also outline how we simulate synthetic baseline surveys with desired average properties.

### Combining the dynamical and statistical modelling

The next step is to parameterise the transmission model for each cluster. Here, locally acquired cluster-specific information would ideally be used, such as dominant mosquito species, ITN coverage, ITN effectiveness (determined by level of mosquito insecticide resistance and ITN class), and the presence of other interventions against malaria. This information provides greater realism to the model predictions and is used to estimate the underlying transmission intensity in the cluster—quantified as the annual entomological inoculation rate in the absence of control interventions—using the cali R package.^17^

Clusters are assigned to the treatment arms using restricted randomisation. In this example, we restrict by malaria prevalence: in practice, other cluster-level information could be introduced here. Information on malaria transmission in each cluster (active interventions and their estimated coverage, seasonality of transmission, etc) can be used to inform the transmission modelling. Once the clusters have been randomised to the two trial arms, we run the transmission model for the trial period, specifying whether the trial or control intervention parameterisation should be used for a given cluster. This produces cluster-level predictions of malaria prevalence over time (online supplemental figure 2). We then choose when to carry out a follow-up prevalence survey, and sample m individuals from the defined age group in each cluster on this day to test for malaria parasites. An unpaired t-test is performed on the simulated cluster prevalence estimates to test for a significant difference between the arms. The binomial sampling process is then repeated S times (here S=1000) to estimate statistical power at this timepoint. The choice of the value of S to use is a trade-off between precision and feasibility in terms of required computation time, as discussed in the literature.^18^ The timing of the follow-up survey can be modified and the whole process repeated, if desired. In the modelling presented here, we show follow-up results for each day of the trial period to allow us to see how statistical power changes over the course of the trial. We note that to calculate study power it is not necessary to separately calculate the effect size (obtained *via* $\pi_{0}$ and $\pi_{1}$), $k_{0}$ or $k_{1}$. However, we present the time evolution of these quantities to highlight how they influence the statistical power.

### The malaria transmission model

The transmission modelling carried out here is done using the malariasimulation R package,^19^ which is a stochastic individual-based implementation of the malaria model developed at Imperial College London (Griffin *et al*^20^). It incorporates exposure-driven immunity to malaria, heterogeneous mosquito biting, and allows the inclusion of a range of interventions against malaria, such as ITNs, SMC, IRS and malaria vaccines. The core model is outlined in detail elsewhere,^21 22^ but we will discuss the details of the interventions modelled here, and how they are characterised in the transmission model. In the model, the presence of an ITN acts to reduce the proportion of attempted mosquito feeding events that result in a blood-meal being obtained. If a mosquito is prevented from feeding, it is either repelled or killed due to the action of the insecticide. The likelihood of each event is determined by the type of net and the level of pyrethroid resistance present in the mosquito population. The values of these parameters are typically estimated using data from experimental hut trials. We use values estimated by Churcher *et al*^23^ who used statistical modelling to predict ITN efficacy at different levels of pyrethroid resistance, for various ITN classes. Throughout this work we assume moderate levels of pyrethroid resistance (40% survival of mosquitoes in the WHO bioassay^24^) in all clusters. To model the administration of SMC, we followed Thompson *et al*,^25 26^ who used clinical trial data to fit a time-varying model for the protection against malaria provided by SP-AQ.^27^ During each transmission season, children received four rounds of SP-AQ, 30 days apart. For this intervention, we used a coverage of 75% in the target age group (3–59 months) in clusters scheduled to receive SMC. This value is motivated by values achieved in current programmes,^28^ though it is important to note that our definition of coverage corresponds to receiving all doses in each round. For simplicity, SMC is introduced as a new intervention at the start of the simulation, although this need not be the case.

## Supplementary material

10.1136/bmjgh-2026-024344 online supplemental file 1

## Data Availability

Data sharing not applicable as no datasets generated and/or analysed for this study.
