# Supplementary material for: Combining statistical and dynamical modelling to guide the design of cluster randomised trials for malaria
Source: BMJ Glob Health. 2026 Aug 28;11(8):e024344. doi: 10.1136/bmjgh-2026-024344 (PMC13536055; doi:10.1136/bmjgh-2026-024344)
Supplement: Supplementary file 1 [file bmjgh-11-8-s001.pdf]

Combining statistical and dynamical modelling to guide the design of cluster randomised trials for malaria

Joseph D. Challenger[1], Joseph Biggs[2], Janetta Skarp[1], Jackie Cook[2], Thomas S. Churcher[1]

1. Medical Research Council Centre for Global Infections Disease Analysis, Department of Infectious Disease Epidemiology, School of Public Health, Imperial College London, London, UK

2. International Statistics and Epidemiology Group, Department of Infectious Disease Epidemiology and International Health, London School of Hygiene and Tropical Medicine (LSHTM), London, UK

SUPPLEMENTARY MATERIALS

This document contains:

1. **Supplementary Methods**
2. **Supplementary Figures 1-8**
3. **Supplementary Tables 1&2**

All references are numbered as per the main text.

Equation numbering follows on from the main text

Supplementary Methods

Estimating cluster-level prevalences

Although it can be difficult to anticipate the value of k in advance of a CRT, the baseline prevalence survey can be used to generate an estimated value. However, it is important to appreciate that two sources of variation are present: binomial sampling variation and variation due to intrinsic heterogeneity between clusters. To estimate k, we must account for the sampling variation. Following Ref. [1], we write:

$$E(s^2) = \sigma_B^2 + \frac{\pi(1-\pi)}{m_H}. \qquad (2)$$

This indicates that the expected value of the empirical variance of cluster prevalences can be decomposed into two terms: the between-cluster variance (σ_B^2), from which we can calculate k, and the binomial sampling variation. Here π is the mean prevalence across the clusters and m_H is the harmonic mean of the sample sizes used in each cluster, which reduces to m if the same sample size is used in each cluster.

The two sources of variability in the baseline survey complicates the parameterisation of the transmission dynamics model, as we want to calibrate the model separately for each cluster. If we calibrated the model to the empirical clusters prevalences (n_j/m_j, where n_i is the number of malaria-positive individuals in cluster j), we would inflate the between-cluster variability predicted by the model, due to the presence of the binomial sampling variation. Instead we proceed by fitting a linear mixed-effects model to the baseline survey data. Denoting the malaria status of individual i in cluster j as x_{ij} ($x_{ij} = 0$ indicating a negative test result, x_{ij}=1 indicating a positive test result), we write the model as:

$$x_{ij} = \alpha + \beta_j + \epsilon_{ij}. \qquad (3)$$

Here α is the model intercept, β_j is the cluster-specific random effect, and ϵ_{ij} is the residual term. From the fitted model we extract the model-predicted prevalence for each cluster: this is equal to $\alpha + \beta_j$ for cluster j.

Supplementary Figure 1 shows a simulated baseline survey for a trial with $c = 20$. We show the model-predicted prevalences for each cluster (red points), compared to the raw values (black points). The model is more sceptical of outlying data points and acts to pull the prevalence of the cluster towards the overall mean value. We shall use these model-predicted prevalences to inform the transmission modelling.

As an aside, we also briefly present an alternative way to describe between-cluster variability that appears elsewhere in the literature. This description uses a measure known as the intracluster correlation coefficient, ρ [1]. It can be written as:

$$\rho = \frac{\sigma_B^2}{\sigma^2} = \frac{\sigma_B^2}{\sigma_W^2 + \sigma_B^2}. \qquad (4)$$

Here, σ_B^2 is the between-cluster variance and σ^2 is the overall variance, for an individual selected at random from any cluster. This latter term can be written as a sum of the between-cluster variance and the within-cluster variance, σ_W^2. Using $k = \frac{\sigma_B}{\pi}$ and following Ref [1], we can write:

$$\rho = \frac{k^2\pi}{1-\pi}. \qquad (5)$$

Note this relationship is valid for binary outcomes. Hence, for the trials considered here, we can move between the two formalisms, if desired. We can also use this relationship to express the within-cluster variability, σ_W^2, in terms of k, σ_B^2, and π.

Simulating baseline surveys

We now outline how to simulate baseline prevalence surveys, sampled from a distribution with a specified values of π and k. We first draw values for the true cluster prevalences from a distribution with mean π and variance $v = \sigma^2$. We draw these from a beta distribution $B(\alpha, \beta)$ to ensure cluster level prevalence lie between 0 and 1, setting $\alpha = \pi\left[\frac{\pi(1-\pi)}{v} - 1\right]$ and $\beta = (1-\pi)\left[\frac{\pi(1-\pi)}{v} - 1\right]$. We make $2c$ draws from this distribution *i.e.* one per cluster. We then use each true cluster prevalence to generate the survey prevalence by generating a binomial sample of size m.

Supplementary Figure 1: Illustration of the model-predicted cluster-level prevalences. Here we simulate cluster-level data (black dots) for a given mean prevalence (π=0.1, dotted grey line), between-cluster heterogeneity (k=0.3) and sample size (m=50). The heterogeneity observed in this data is generated by a mixture of intrinsic heterogeneity and binomial sampling. By fitting a linear mixed-effects model, we obtain predictions for the true cluster-level prevalences (red points). The grey arrows emphasise how the model predictions are pulled in towards the average (empirical) prevalence observed across the clusters (solid grey horizontal line). The model-predicted prevalences are used as inputs for the dynamical transmission modelling.

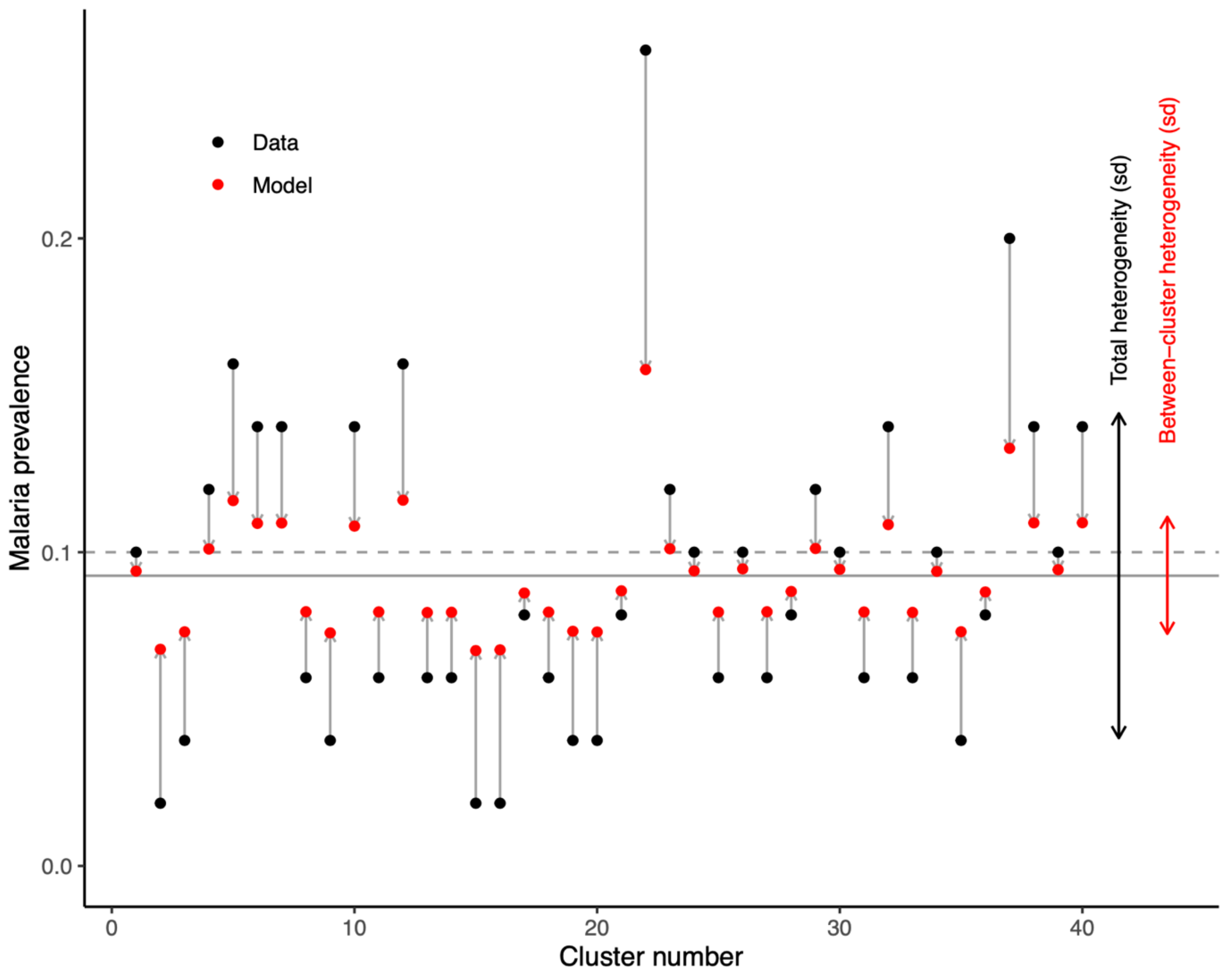

Supplementary Figure 2: Cluster-level dynamics of a cluster-randomised trial (CRT). Here we show results for a simulated CRT with 20 clusters per arm. The black dots are the data points from the baseline prevalence survey (error bars show the 95% CIs). The baseline survey was simulated to have an average prevalence of 0.1 and a between-cluster heterogeneity of 0.3. The model-predicted prevalences, calculated to account for the sampling variation in the baseline prevalence survey, are also shown (magenta triangles). The model-predicted values are used to calibrate the transmission model in each cluster and randomise each cluster to a treatment arm. During the trial period, follow-up surveys are generated by performing binomial sampling in each cluster. An unpaired *t* test can then be performed to assess whether there is a significant difference between the trial arms.

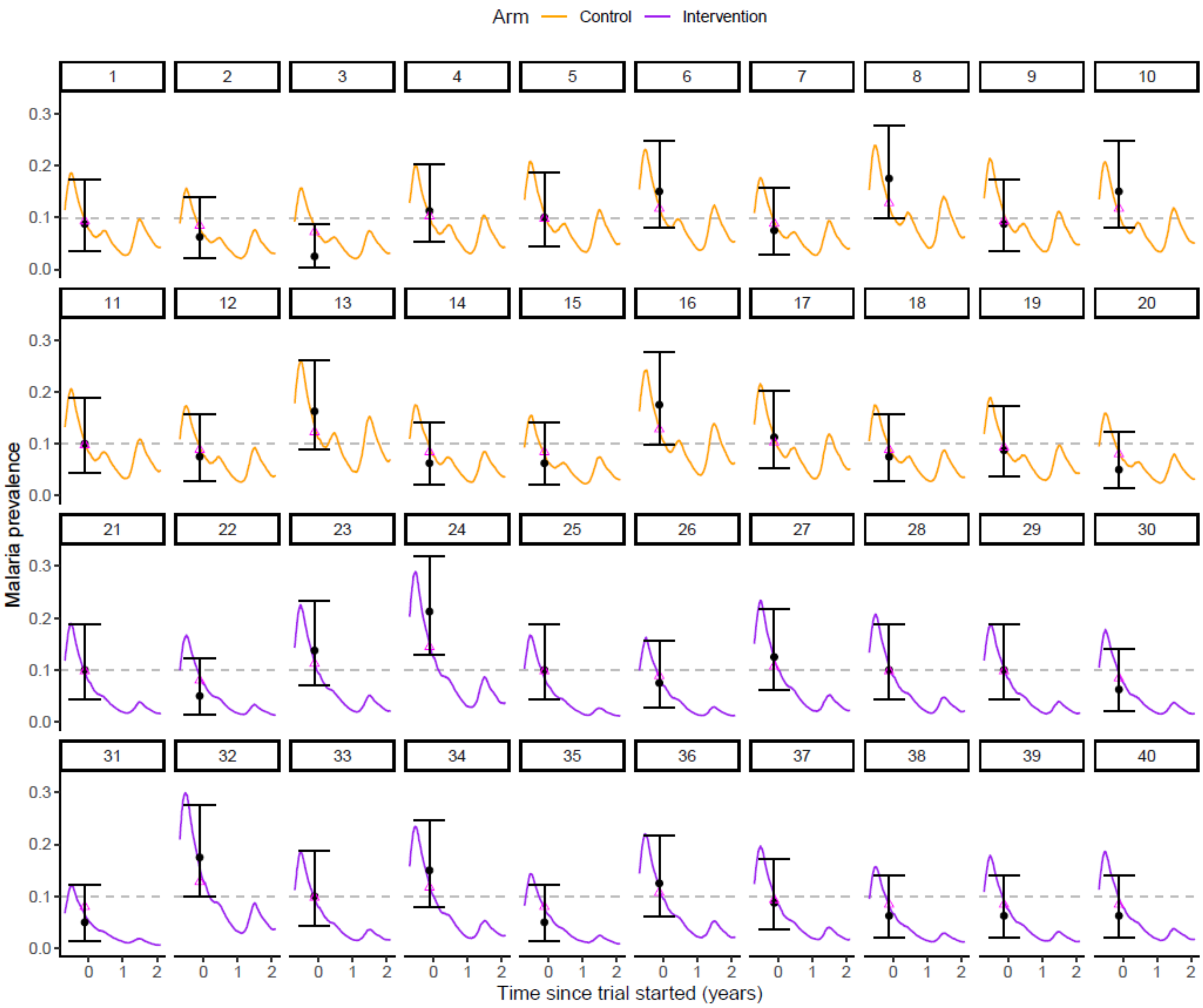

Supplementary Figure 3: Illustration of varying the timing of the follow-up survey. In the results shown in Figures 3 and 4, we vary the average endemicity across the trial sites and the timing of the follow-up survey. Here we illustrate this procedure, for the case where the average prevalence at baseline was 40% in the age-group of interest (2-10 year olds). We show this for both the non-seasonal (panel A) and non-seasonal (panel B) scenarios. The solid vertical line indicates the time of the mass ITN campaign, whilst vertical dashed lines denote the timing of the cross-sectional surveys reported in Figure 3. The vertical dotted line indicates the timing of the baseline survey, carried out six weeks prior to the trial commencing.

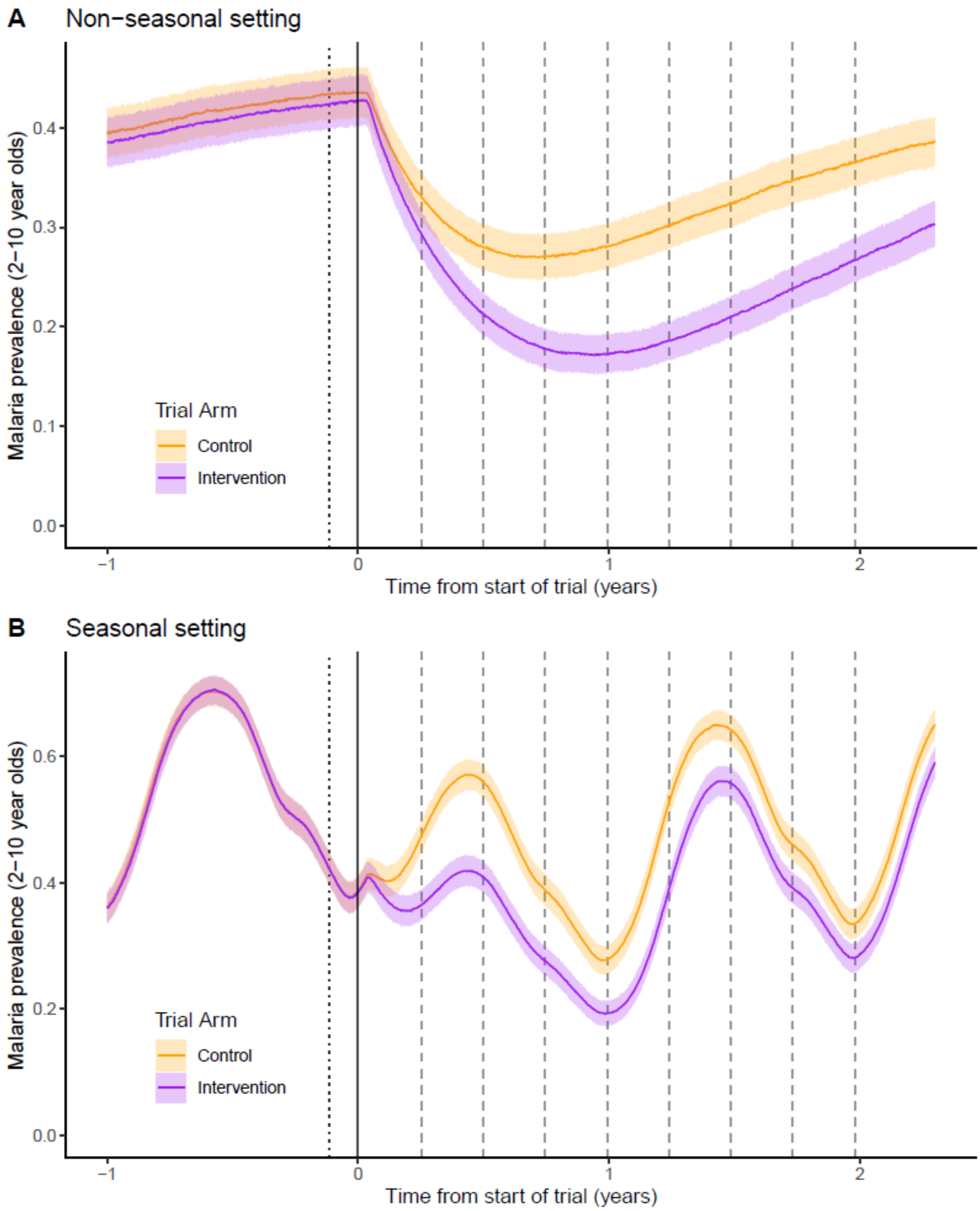

Supplementary Figure 4: Estimating trial outcomes at different levels of malaria endemicity in a non-seasonal setting. Here simulated trial data is assessed at eight different follow-up times, in a trial lasting two years (scenario is illustrated in Supplementary Figure 3A). The mean prevalence in the baseline survey (measured in 2-10 year olds) is varied from 10-45%. Panel A: time-evolution of the achieved effect size at different malaria endemicities. In the first year of the trial, the effect size grows in a similar way at differing endemicities (x axis), but more slowly than in the seasonal example (Figure 3). In the second year of the trial, a large effect size is maintained in low transmission settings, but diminishes in high-transmission settings. This observation is reflected in the power estimates (panel B), indicating that it is more difficult to power these studies at very high transmission intensities. At intermediate transmission intensity (baseline prevalence of 20-25%), >80% power is maintained until the end of the two-year period. At low transmission intensity, we also show results obtained when the expected effect size (relevant for the sample size calculations) is expected to be >30% (see legend of panel B). At variance to the seasonal setting, here an effect size of 40% is well-powered. Statistical power is also influenced by the between-cluster heterogeneity, which is shown in Supplementary Figure 5. The sample size used in each scenario is shown in Supplementary Table 1.

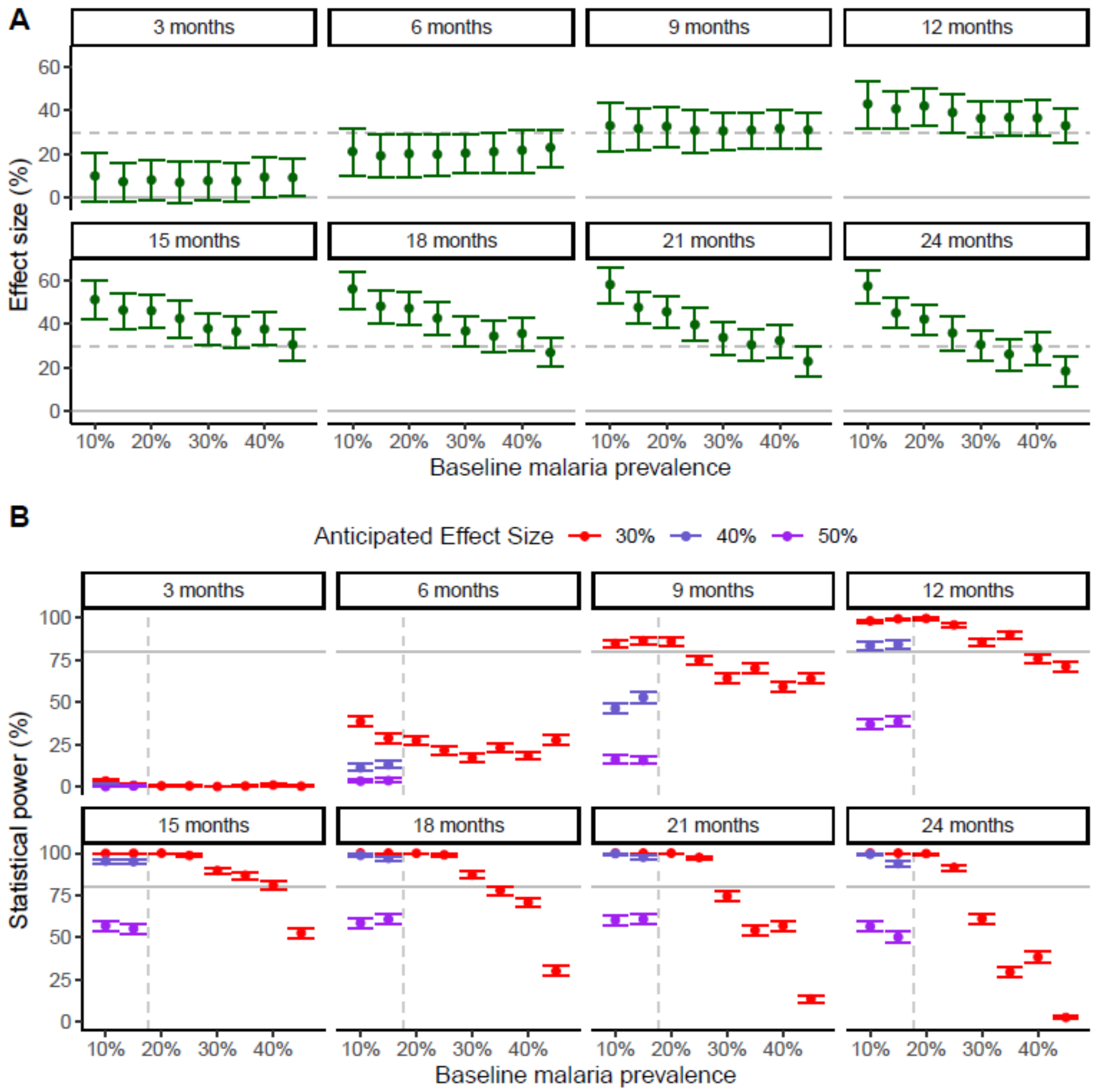

Supplementary Figure 5: Estimating the between-cluster heterogeneity (k) at different levels of malaria endemicity in a non-seasonal setting. Here simulated trial data is assessed at eight different follow-up times, in a trial lasting two years (scenario is illustrated in Supplementary Figure 3A). The average prevalence at baseline is varied from 10-45% (in 2-10 year olds). The value of k at baseline varies with prevalence, as shown in Supplementary Table 1, with higher values used at lower prevalence. Three months into the trial, the average values of k (points) are similar in each arm. Later in the trial, however, k increases as prevalence decreases, especially in the intervention arm. The very high values of k observed in low transmission settings towards the end of the trial period make it harder to power these studies, especially when a large effect size was expected (Figure 4B). The error bars indicate the central 95% range of estimated k values at each time point, generated by simulating 1000 follow-up surveys. The horizontal grey line ($k = 0.3$) is shown as a reference.

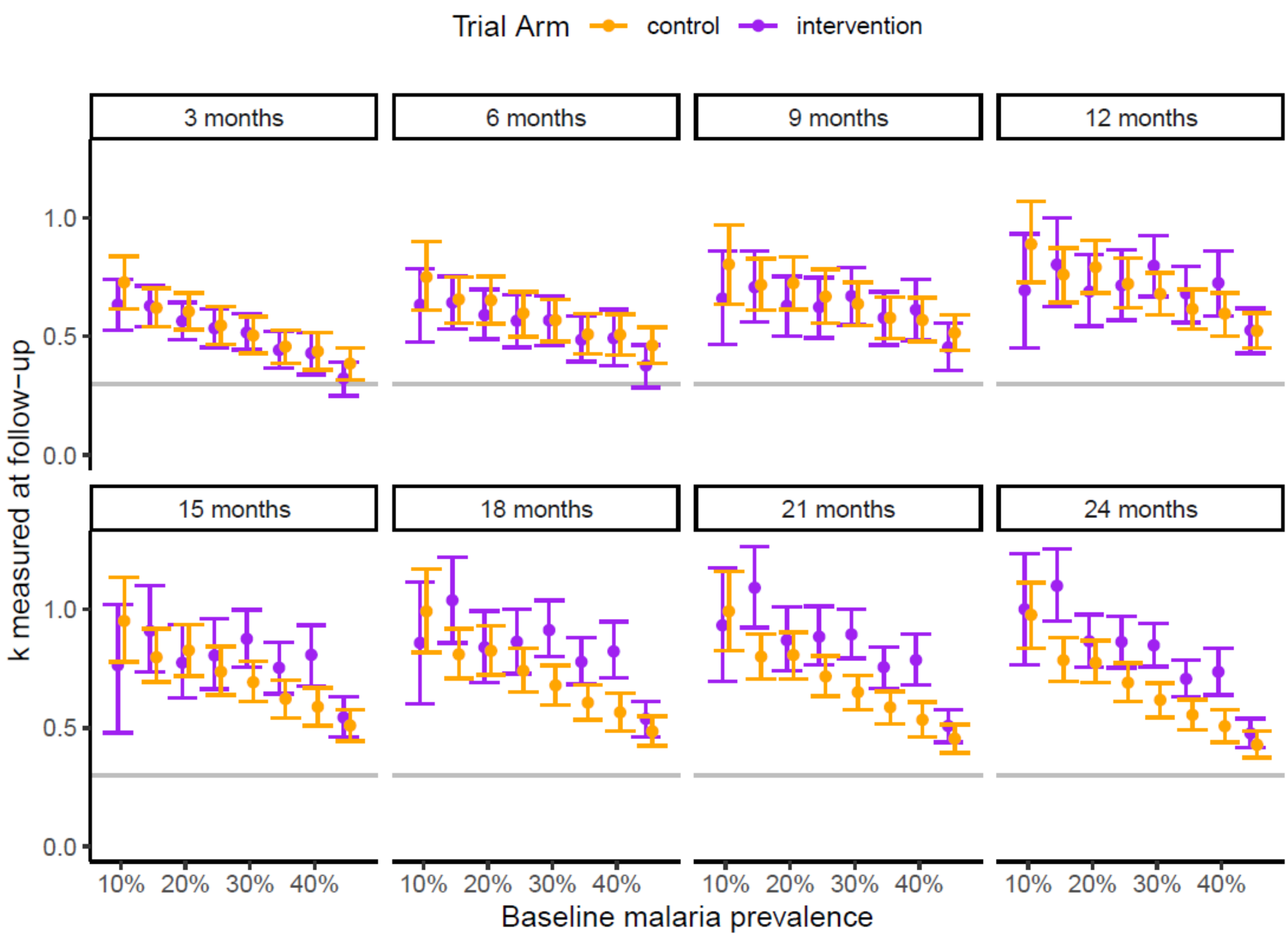

Supplementary Figure 6: Estimating trial outcomes at different levels of malaria endemicity in a seasonal setting, with different parameter values used compared to the main text. Panel A: here we vary the value of malaria prevalence at baseline (x axis), whilst fixing all other parameters (expected effect size, number of clusters, number of people sampled per cluster, $k = 0.3$). Here, the achieved effect size, as predicted by the transmission modelling, is the dominant factor, leading to high statistical power at low and intermediate values of baseline prevalence, and inadequate power at high transmission, especially at or above a baseline prevalence of 35%. Panel B: here we vary the value of malaria prevalence at baseline, and fix the value of k at 0.3. We then vary the number of clusters per arm to achieve 80% power (see Supplementary Table 2). Again, we find low power in high transmission settings, due to the effect size. Power is higher in low-transmission settings, even when the expected effect size was 40%. However, power was lower when an expected effect size of 50% was used. Measurements taken during the malaria season are highlighted with the light green shading.

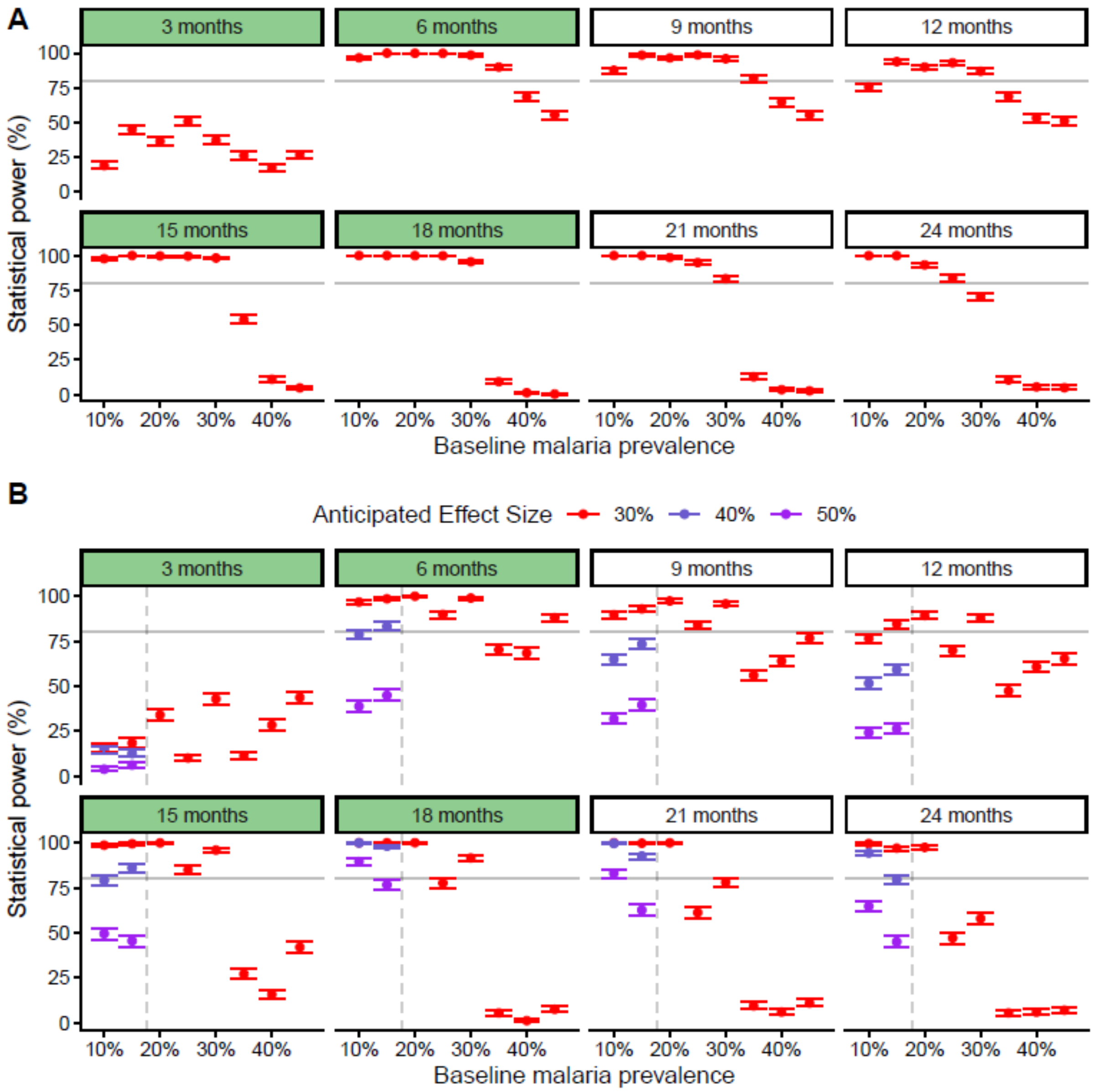

Supplementary Figure 7: Estimating trial outcomes at different levels of malaria endemicity in a non-seasonal setting, with different parameter values used compared to the main text. Panel A: here we vary the value of malaria prevalence at baseline (x axis), whilst fixing all other parameters (expected effect size, number of clusters, number of people sampled per cluster, k=0.3). Here, the achieved effect size, as predicted by the transmission modelling, is the dominant factor, leading to high statistical power at low and intermediate values of baseline prevalence, and inadequate power at high transmission, especially at or above a baseline prevalence of 35%. Panel B: here we vary the value of malaria prevalence at baseline, and fix the value of k at 0.3. We then vary the number of clusters per arm to achieve 80% power (see Supplementary Table 2). Again, we find low power in high transmission settings, due to the effect size. Power is higher in low-transmission settings, even when the expected effect size was 40%. However, power was lower when an expected effect size of 50% was used.

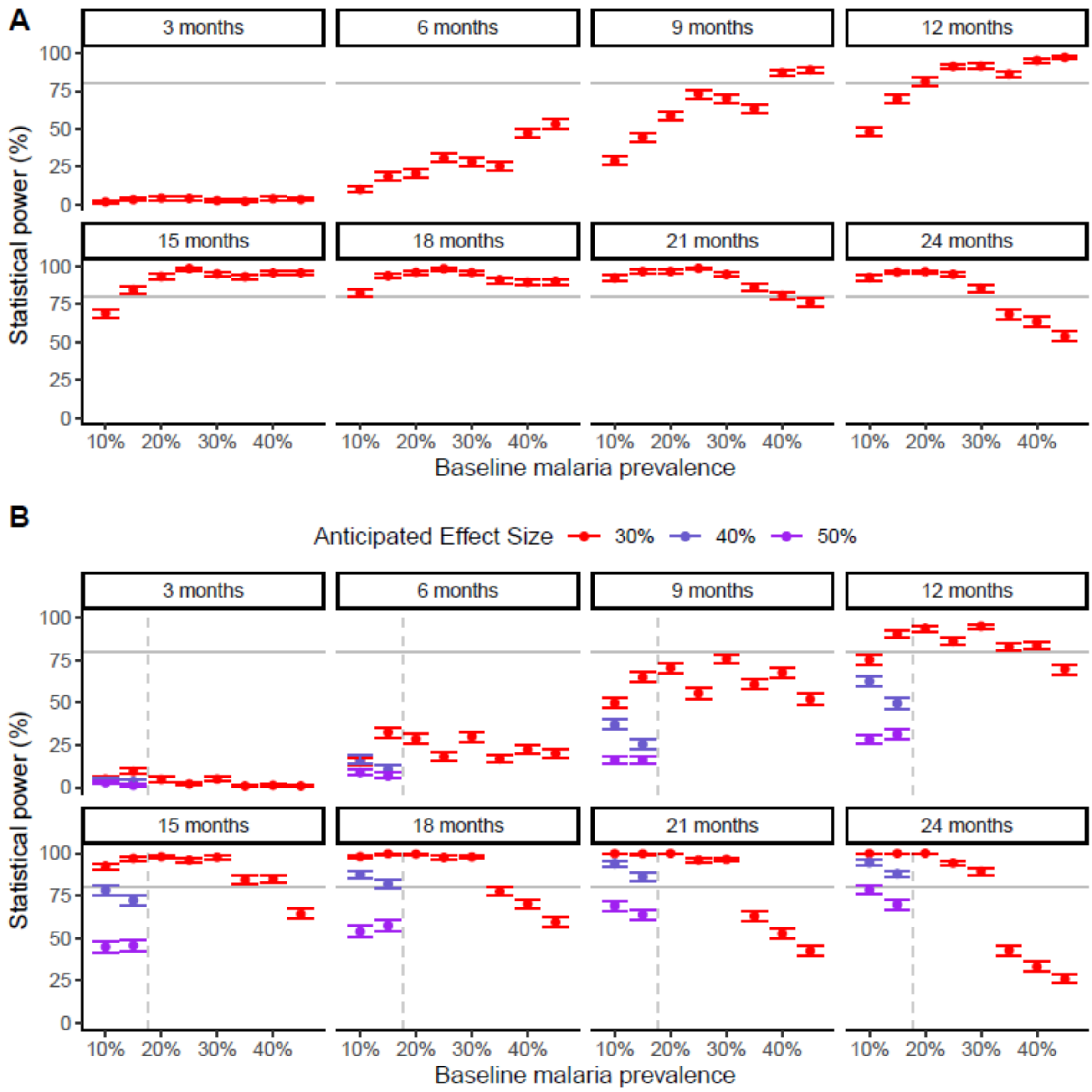

Supplementary Figure 8: The impact of background interventions on cluster randomised trials (CRTs). In Figure 5 we evaluated a pyrethroid-pyrrole ITN against a standard pyrethroid ITN, for the case where young children (aged 3-59 months) were receiving seasonal malaria chemoprevention. Here we perform a sensitivity analysis, in which follow-up is conducted in young children (0.5-5 years old), and in the analysis (unpaired *t* test) the cluster-level data is adjusted for the presence (or absence) of SMC. The purple curve shows the statistical power without the adjustment (curve is identical to that shown in Figure 5), the grey curve shows the power with the adjustment. Adjusting the analysis in this way does increase the study power, but power is still higher in the other age groups examined (Figure 5).

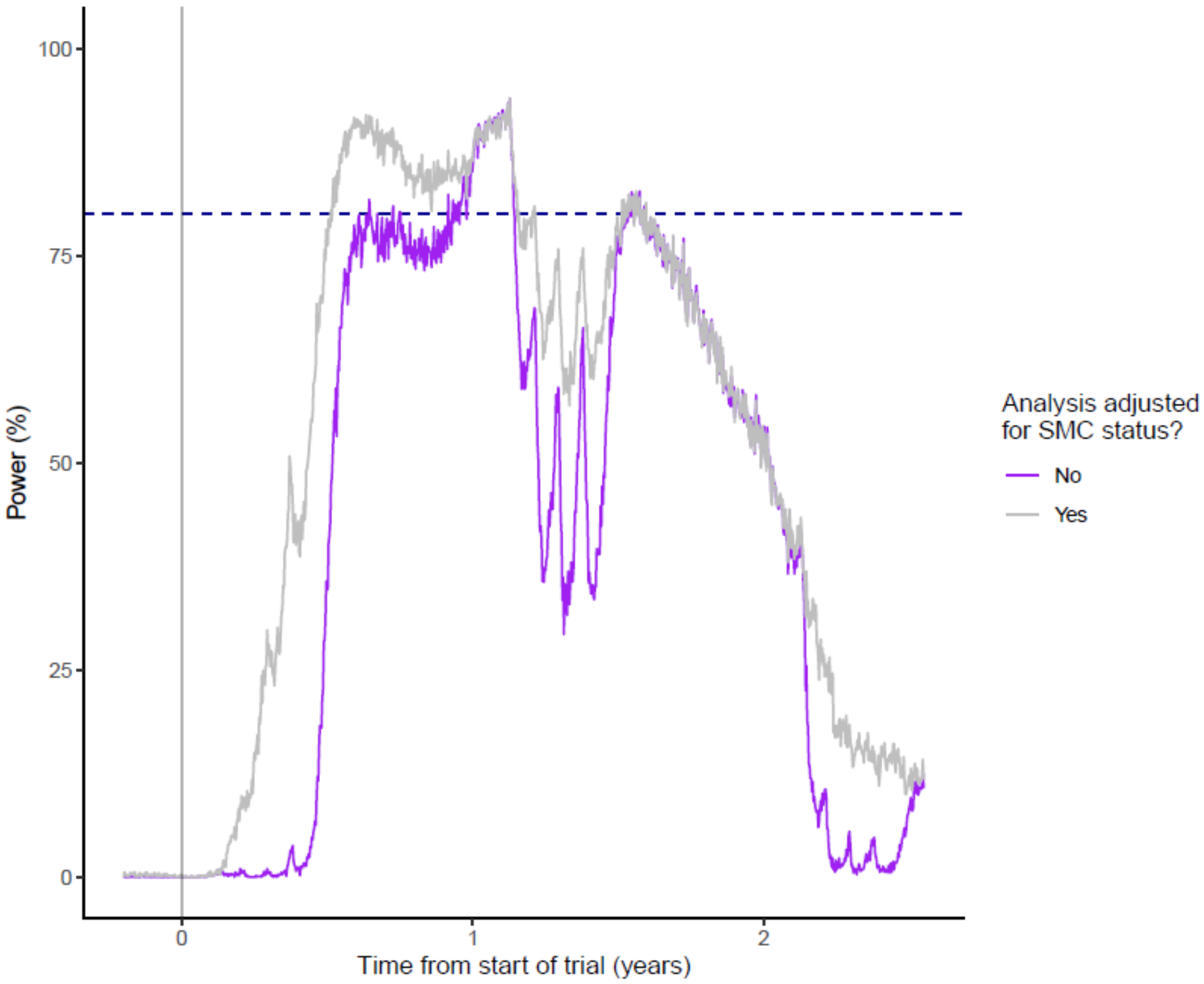

Supplementary Table 1: Sample sizes used when varying the average baseline prevalence for a cluster randomised trial. In these scenarios, the between-cluster heterogeneity present in the baseline prevalence survey was chosen to match the values obtained in a meta-analysis of malaria cluster randomised trials (see Figure 5a in Ref. [3]). The number of people sampled per cluster (m) is held fixed, and the number of clusters per arm, c, is then chosen to obtain 80% power for the desired effect size, using Equation 1.

Baseline prevalence	Number of people sampled per cluster, m (fixed)	Between-cluster heterogeneity, k	Clusters (c) per arm required for 80% power (effect size=30% unless stated)	Total sample size ($2mc$)
10%	50	0.74	99 (52 *for 40% effect size)* ***(31 for 50% effect size)***	9900 *(5200)* ***3100***
15%	50	0.67	76 *(40 for 40% effect size)* ***(24 for 50% effect size)***	7600 *(4000)* ***2400***
20%	50	0.6	60	6000
25%	50	0.54	48	4800
30%	50	0.49	39	3900
35%	50	0.44	32	3200
40%	50	0.4	27	2700
45%	50	0.35	21	2100

Supplementary Table 2: Sample sizes used when varying the average baseline prevalence for a cluster randomised trial. In these scenarios, both the between-cluster heterogeneity (k) and the number of people sampled per cluster (m) are held fixed. The number of clusters per arm (c) is then chosen to obtain 80% power for the desired effect size, using Equation 1.

Baseline prevalence	Number of people sampled per cluster, m (fixed)	Between-cluster heterogeneity, k (fixed)	Clusters per arm (c) required for 80% power (effect size=30% unless stated)	Total sample size ($= 2mc$)
10%	50	0.3	40 (*22 for 40% effect size)* ***(13 for 50% effect size)***	4000 *(2200)*
15%	50	0.3	30 *(17 for 40% effect size)* ***(10 for 50% effect size)***	3000 *(1700)*
20%	50	0.3	25	2500
25%	50	0.3	22	2200
30%	50	0.3	20	2000
35%	50	0.3	19	1900
40%	50	0.3	18	1800
45%	50	0.3	17	1700
